# Antihypertensive Medications Use among Chronic Hemodialysis Patients Visiting the Outpatient Department of Nephrology of a Tertiary Care Centre: A Descriptive Cross-sectional Study

**DOI:** 10.31729/jnma.8095

**Published:** 2023-03-31

**Authors:** Lujaw Ratna Tuladhar, Diwakar Manandhar, Sanjida Ansari, Nabin Khadka, Deepak Regmi, Bijaya Laxmi Shrestha

**Affiliations:** 1 Department of Pharmacology, Nepal Medical College and Teaching Hospital, Kathmandu, Nepal; 2 Department of Internal Medicine, Nepal Medical College and Teaching Hospital, Kathmandu, Nepal; 3 Nepal Medical College and Teaching Hospital, Kathmandu, Nepal

**Keywords:** *anti-hypertensive drugs*, *hemodialysis*, *prevalence*

## Abstract

**Introduction:**

Anti-hypertensive medications are prescribed for the management of high blood pressure which is the leading cause of mortality in chronic hemodialysis patients. The objective of our study was to find out the prevalence of anti-hypertensive medication use among chronic hemodialysis patients visiting the outpatient Department of Nephrology of a tertiary care centre.

**Methods:**

This was a descriptive cross-sectional study conducted among chronic hemodialysis patients visiting the Department of Nephrology of a tertiary care centre from 2 April 2022 to 30 September 2022. Ethical approval was taken from the Institutional Review Committee (Reference number: 062-078/079). A convenience sampling method was used. Point estimate and 90% Confidence Interval were calculated.

**Results:**

The prevalence of anti-hypertensive medications use was present in 102 (97.14%) (93.95-100, 90% Confidence Interval) patient undergoing hemodialysis. The three common drugs prescribed for hypertensive patients were amlodipine 79 (77.45%), torsemide 59 (57.84%), and prazosin 48 (47.05%).

**Conclusions:**

The prevalence of antihypertensive medication use among patients undergoing hemodialysis was higher than other similar studies done in similar settings.

## INTRODUCTION

Hypertension is one of the leading causes of mortality (>50%) in chronic hemodialysis patients.^1^ Antihypertensive medication like calcium channel blockers (CCB-amlodipine), alpha-blockers (prazosin), betablockers (atenolol, bisoprolol, carvedilol, metoprolol), angiotensin-converting enzyme inhibitors (ACEI), angiotensin receptor blockers (ARB), centrally acting alpha-2 agonists (clonidine), diuretics (torsemide), vasodilators, and direct renin inhibitors (aliskiren) are prescribed for the management of high blood pressure.^2,3^

The practice pattern of anti-hypertensive medication may vary in chronic hemodialysis patients as it can impair the drug's pharmacokinetic properties.^4^

The objective of our study was to find out the prevalence of anti-hypertensive medication use among chronic hemodialysis patients visiting the outpatient Department of Nephrology of a tertiary care centre.

## METHODS

This was a descriptive cross-sectional study conducted on chronic hemodialysis patients visiting the Department of Nephrology at Nepal Medical College and Teaching Hospital (NMCTH), Kathmandu, Nepal. The study commenced on 2 April 2022 to 30 September 2022 for 6 months. Ethical approval was taken from the Institutional Review Committee (Reference number: 062-078/079). Patients who visited the Nephrology Department for hemodialysis and who were willing to participate voluntarily in the study were included. Drugs other than anti-hypertensive medication and follow-up patients were also excluded from this study. A convenience sampling method was used. The sample size was calculated by using the following formula:


$$\begin{array}{ll} \text{n} & ={\text{Z}}^{2}\times \frac{\text{p}\times \text{q}}{{\text{e}}^{\text{2}}}\\ & =1.96^{2}\times \frac{0.50\times 0.50}{0.10^{2}}\\ & =97 \end{array}$$

Where,

n = minimum required sample size
Z = 1.96 at 90% Confidence Interval (CI)
p = prevalence taken as 50% for maximum sample size calculation
q = 1-p
e = margin of error, 10%

The calculated minimum required sample size was 97. However, 105 patients were included in the study.

Data on the patient's details and anti-hypertensive medication profile were collected from the dialysis unit of the Department of Nephrology by the researcher by interviewing individually through the preformed self-constructed questionnaire. The predesigned questionnaire was used. Anti-hypertensive drugs used for the management of complications during dialysis were also reported.

Data were entered and analyzed with IBM SPSS Statistics 16.0. Point estimate and 90% CI were calculated.

## RESULTS

The prevalence of anti-hypertensive medication use was 102 (97.14%) (93.95-100, 90% CI) among patients undergoing chronic hemodialysis. There were a total of 60 (58.82%) males and 42 (41.17%) females. The patient's age group was ranging from 21 to 81 years with a mean age of 45.75±14.91 years. The three common antihypertensive medications prescribed for patients on chronic hemodialysis patients were amlodipine 79 (77.45%), torsemide 59 (57.84%), and prazosin 48 (47.05%) (Table 1).

**Table 1:** The pattern of antihypertensive medication in chronic hemodialysis patients (n= 102).

Medication group	n (%)
**Calcium channel blocker**	
Amlodipine	79 (77.45)
**Diuretic**	
Torsemide	59 (57.84)
**Alpha blocker**	
Prazosin	48 (47.05)
**Beta-blockers**	
Metoprolol	26 (25.49)
Bisoprolol	1 (0.98)
Carvedilol	4 (3.92)
Atenolol	2 (1.96)
**Alpha-2 agonist**	
Clonidine	20 (19.60)

During dialysis, intradialytic hypotension was reported in 4 (3.92%) while intradialytic hypertension was reported in 18 (17.64%) of the patients. Intradialytic hypertension was managed with centrally acting alpha-2 agonists (clonidine) and intradialytic hypotension was managed with crystalloid fluids like normal saline and IV dextrose(Table 2).

**Table 2:** Management of intradialytic hypotension and intradialytic hypertension (n= 102).

**Intradialytic hypotension management**	
**Crystalloid fluid and medication**	**n (%)**
Normal saline	1 (0.98)
IV dextrose	1 (0.98)
IV dextrose + normal saline	2 (1.96)
**Intradialytic hypertension management**	
Clonidine	18 (17.64)

## DISCUSSION

This study found the prevalence of anti-hypertensive medications used in chronic hemodialysis patients was 97.14%. A study conducted in Kolkata reported that the drugs prescribed to the majority of hemodialysis patients were anti-hypertensive medicines (23.41%).^5^ Among the anti-hypertensive medicines, diuretics (9.29%), calcium channel blockers (5.92%), and alphablockers (2.91%) were commonly prescribed.^5^ In our study, we observed that calcium channel blocker (amlodipine- 77.5%) was frequently prescribed to the patients undergoing hemodialysis followed by diuretics (torsemide- 57.8%) and alpha-blockers (prazosin- 47.1%). Although in the study, diuretics were prescribed the most,^5^ other studies have reported calcium channel blockers as the most common drug to be prescribed.^6,7^

A study reported that CCB has been associated with decreased mortality when compared with alpha-1 blockers, beta-blockers, ACEI, ARB, and nitrates.^8^ Calcium channel blockers are mainly eliminated by hepatic metabolism and can usually be administered in standard dosages in patients with severe renal impairment.^9^ However, some of the CCBs (nifedipine, verapamil, diltiazem) have active metabolites that may require renal clearance.^4^ CCBs have also been found to reduce proteinuria and slow the progression of renal insufficiency independently of their blood pressure lowering effect.^10,11^ Similarly, a-blocker have also been found to have renoprotective action.^12^ In our study, we did not observe any ACE Inhibitors prescribed to our patients. Although ACE inhibitors and ARB slows the progression of renal failure; they were not prescribed for patients with ESRD as these medications can lead to hyperkalemia.^6^

The thiazide group of diuretics is less efficacious in a patient with advanced renal disease. The efficacy of furosemide also decreases in a patient with advanced CKD and therefore the dose needs to be increased with a subsequent increase in its adverse effect i.e., ototoxicity. However, torsemide was found to be effective and its pharmacokinetics parameter remains unchanged even in advanced kidney disease.^13^ Torsemide was also found to have renoprotective action.^14^ Potassium-sparing diuretics should be avoided in patients with eGFR less than 35-45 ml/min due to a marked increase in hyperkalemia.^4^

End-stage renal disease (ESRD) patients are susceptible to cardiac failure.^15^ Therefore, beta blockers are prescribed for hemodialysis patients.^16^ Beta blockers have reduced the risk of cardiovascular disease and favour cardioprotective action among hemodialysis patients.^17^ In our study, beta-blockers like metoprolol-25.5%, carvedilol-3.9%, atenolol-2% and bisoprolol-1% were prescribed to hemodialysis patients. Another study observed the use of the majority of beta blockers like carvedilol, metoprolol, atenolol, bisoprolol, and acebutolol in chronic hemodialysis patients.^18^

β-Blockers can reduce renal blood flow and adversely affect renal function in patients with severe renal impairment. The dose of β-blockers should be reduced especially if eliminated through the renal route, e.g. atenolol. Renally excreted β-blockers (especially atenolol) may accumulate in ESRD and cause profound bradycardia and subsequent hypotension and collapse. β-blockers like metoprolol are preferred.^9^ β-blockers like metoprolol and carvedilol are eliminated by the liver and do not require dose adjustment in renal failure while hydrophilic beta blockers like atenolol and bisoprolol need a dose adjustment.^4^

Although hemodialysis is a life-sustaining procedure for end-stage kidney disease (ESKD) patients, there is a tendency for blood pressure (BP) to change frequently during hemodialysis.^19^ Two of the complication that occurs during hemodialysis are intra-dialytic hypotension^20^ and intra-dialytic hypertension.^21^

Intradialytic hypertension as defined by the National Kidney Foundation's Kidney Disease Outcomes Quality Initiative [KDOQI] as a post-dialysis BP >130/80 mmHg).^22^ Patients with intradialytic hypertension are at high risk for renal failure and antihypertensive medications should be prescribed.^23^ Intradialytic hypertension occurs regularly in 10-15% of hemodialysis patients.^22^

In our study, intradialytic hypertension was reported in 18 (17.6%) of the patients. The use of the highly dialysable drug like metoprolol was associated with a high risk of intradialytic hypertension.^24^ In our study, metoprolol was used in 26 (25.49%) which could have led to a higher rate of intradialytic hypertension. In our study, intradialytic hypertension was managed with clonidine. Intradialytic hypertension can be prevented by the use of less dialyzable drugs like carvedilol.^19^ Clonidine has been found to be effective at reducing systolic blood pressure in hemodialysis patients.^25^ Only 5% of clonidine is removed by hemodialysis.^4^

Intradialytic hypotension occurs in 10-12% of the patients. An intradialytic systolic BP of less than 90mm Hg is associated with mortality.^26^ In our study, we observed intradialytic hypotension was observed in 4 (3.9%) of the patients and was managed with normal saline and IV dextrose. Intradialytic hypotension was more common in those on carvedilol (a poorly dialyzable P-blocker) compared with those on metoprolol (a highly dialysable P-blocker).^18^ In our study, the poorly dialysable drug carvedilol was used in 4 (3.92%) which coincides with the number of intradialytic hypotension cases.

One of the limitations of this study was that the patient who had come for hemodialysis could not recall the names of the medications that were consumed. Such patients had to be followed up on the next visit to reconfirm the name of the medication from their record file. During the dialysis procedure, a few patients collapsed and were therefore excluded from our study.

## CONCLUSIONS

The prevalence of antihypertensive medication was found to be higher than in other studies done in similar settings. Anti-hypertensive medications were prescribed for both hypertensive and non-hypertensive patients on chronic hemodialysis which could be due to renoprotection action. We did not observe the use of newer anti-hypertensive medications which could be due to a lack of efficacy and safety in hemodialysis patients. We also would like to suggest the use of an alternative drug to metoprolol as it could be associated with a higher rate of intradialytic hypertension.
